# Evidence for dysbiosis in the gut microbiome of patients with systemic mastocytosis

**DOI:** 10.1016/j.jacig.2025.100578

**Published:** 2025-10-09

**Authors:** Lauren E. Krausfeldt, Vivian Cao, Richard Rodrigues, Wendy A. Henderson, Robin Eisch, Linda M. Scott, Dean D. Metcalfe, Hirsh D. Komarow

**Affiliations:** a Bioinformatics and Computational Biosciences Branch, Office of Cyber Infrastructure and Computational Biology, National Institute of Allergy and Infectious Diseases, National Institutes of Health, Bethesda, Md; b Mast Cell Biology Section, Laboratory of Allergic Diseases, National Institute of Allergy and Infectious Diseases, National Institutes of Health, Bethesda, Md; c University of Pittsburgh School of Medicine, Pittsburgh, Pa; d Microbiome and Genetics Core, Laboratory of Integrative Cancer Immunology, Center for Cancer Research, National Cancer Institute, Bethesda, Md; e Basic Science Program, Frederick National Laboratory for Cancer Research, Frederick, Md; f Digestive Disorders Unit, National Institute of Nursing, National Institutes of Health, Bethesda, Md; g Department of Biobehavioral Health Sciences, University of Pennsylvania, Philadelphia, Pa

**Keywords:** Systemic mastocytosis, tryptase, KIT D816V, mast cell disease, mast cells, gut microbiome, dysbiosis, short-chain fatty acids, Firmicutes, Bacteroidetes

## Abstract

**Background:**

Limited research studies have investigated the role of the gut microbiome in systemic mastocytosis (SM), which is characterized by an aberrant expansion of clonal mast cells in specific tissues including the skin, marrow, liver, and the gastrointestinal tract.

**Objectives:**

We sought to investigate the relationship between the intestinal microbiome and clinical manifestations of SM.

**Methods:**

The V4 region of the 16S rRNA gene was sequenced from stool samples of 22 patients with SM and 9 healthy controls. Microbial community composition, diversity, and functional genes inferred from 16S rRNA gene sequences were analyzed. ClinicalTrials.gov Identifier NCT00044122.

**Results:**

Changes in microbial community composition were associated with SM, KIT D816V, and tryptase (PERMANOVA, *P* = .004, *P* = .05, *P* = .005, respectively). The differences with SM were driven by the composition of Firmicutes (*P* = .04) and an increase in Bacteroidetes abundance (*P* = .04). Predicted functions of the gut microbiome suggested that there were differences in metabolite profiles, including short-chain fatty acids, increased virulence factors, and decreased bacterial defense mechanisms in patients with SM. Dietary components were associated with symptoms, quality of life, and markers of mast cell activation and inflammation, as well as changes in microbial composition and predicted function in patients with SM.

**Conclusions:**

Dysbiosis of the gut microbiome is evident in patients with SM and is seemingly associated with mast cell activation. In addition, diet may further alter microbial composition and metabolism in the gut of patients with SM.

Systemic mastocytosis (SM) is defined by an aberrant expansion of clonal mast cells that is associated with an increase in release of mast cell–derived mediators.^1^ As a result, patients with SM present with an array of manifestations involving various organ systems including the skin (maculopapular cutaneous mastocytosis), skeletal system (osteoporosis, pain, fragility fractures), liver (organomegaly), and gastrointestinal (GI) tract (abdominal pain, diarrhea, gastroesophageal reflux disease). The manifestations of SM are due to mast cell infiltration into various tissues associated with release of mast cell mediators.^2^ GI symptoms are among the most prominent manifestations of SM, with 60% to 80% of patients with SM experiencing them.^3^ Patients with SM are also at a higher risk for severe allergic reactions, including anaphylaxis.^4^

The impact of the gut microbiome in human health and disease is well accepted.^5^ The gut microbiome itself is composed of commensal microorganisms that interact with the host. They protect the host from pathogens, harvest dietary energy with digestive and metabolic enzymes, and ferment undigested food.^6^^,^^7^ Byproducts of these processes and other microbial metabolites facilitate cross-talk between the gut microbiome and the host. The gut microbiome is also essential for activation of the host immune response and is important in maintaining gut homeostasis and intestinal barrier integrity.^6^^,^^8^ These interactions regulate functions across multiple tissues of the hepatic, nervous, GI, and musculoskeletal systems.^9^, ^10^, ^11^ Examination of the dysbiosis of the gut microbiome can provide insights into the mechanistic cause or effects of a disease, while identifying therapeutics that modulate the gut microbiome, such as oral probiotics, has shown promise for attenuating a disease process.^12^

Mast cells can affect the homeostasis of the gut microbiome in several ways.^13^ First, by interacting with other facets of the immune system that regulate inflammation along with the gut microbiome, they maintain gut barrier integrity.^14^ Second, overactivity of mast cells can influence diseases whose symptoms overlap with those reported by patients with SM. Examples include irritable bowel disorders,^15^ local (urticaria) and systemic (anaphylaxis) allergic reactions, and bone metabolism and disease,^16^ all of which have been associated with dysbiosis.^17^ In addition, evidence suggests that the gut microbiome recruits GI mast cells, influences their maturation, and interacts with these cells both directly and indirectly.^13^^,^^18^^,^^19^ Thus, it is reasonable to predict that such interactions could similarly influence pathology and manifestations of disease in SM.

The goal of this study was thus to characterize the gut microbiome in patients with SM, focusing on differences in the gut microbiome composition, diversity, and predicted metabolism compared with healthy controls (HCs); and relate such findings to clinical aspects of disease including symptoms, markers of inflammation and mast cell activation, and nutrition.

## Methods

Stool samples from 22 patients with indolent SM and 9 HCs were collected during an outpatient clinic visit to the National Institutes of Health Clinical Center following informed consent and enrollment on National Institutes of Health Institutional Review Board–approved protocol (# 02-I-0027). Samples were stored at −80°C until extraction. Demographic, clinical, and molecular correlates from subjects were collected (Table I; see Table E1 and this article’s Methods section in the Online Repository at www.jaci-global.org). Standard laboratory tests were also performed including complete blood cell count with differential, immunoglobulin levels, hepatic panel, and blood chemistries. A comprehensive nutritional analysis was performed for each participant, which covered 3 days of food consumption immediately before stool collection to represent the average diet of the individuals (see this article’s Methods section in the Online Repository).

**Table I:** Demographic and clinical information collected for patients with SM and for HCs

Characteristics	SM	HCs
Age (y), median (IQR)	58 (46-64.75)	36 (26.5-53)
Sex, F:M	10:12	6:3
Race		
Asian	0 of 22 (0%)	2 of 9 (22.2%)
Black or African American	2 of 22 (9.1%)	2 of 9 (22.2%)
White	20 of 22 (90.9%)	5 of 9 (55.6%)
Weight (kg), median (IQR)	88.90 (70.69-101.3)	
BMI, median (IQR)	27.85 (24.53-34.23)	
Mc-QoL, median (IQR)	25.50 (10.50-37.25)	
Symptoms		
Osteopenia	14 of 19 (73.7%)	
Osteoporosis	6 of 20 (30%)	
Joint pain	5 of 22 (20%)	
Bone pain	5 of 20 (20%)	
Abdominal pain	9 of 22 (40.9%)	
GERD	11 of 20 (55%)	
Nausea	5 of 22 (22.7%)	
Diarrhea	12 of 22 (54.5%)	
History of anaphylaxis	11 of 22 (50%)	
Flushing	18 of 22 (81.2%)	
UP	18 of 22 (81.2%)	
Brain fog	1 of 22 (4.5%)	
Headaches	9 of 22 (40.9%)	
Depression	1 of 22 (4.5%)	
Markers of mast cell activation and inflammation, median (IQR)		
D816V, %	0.208 (0.054-2.201)	
Tryptase (ng/mL)	33.05 (19.38-55.23)	3.9 (1.15-5.6)
FCAP (dB/m)	270 (239-331.5)	244 (208-299)
FibroScan score (kPa)	5 (4.20-6.65)	4.8 (4.15-8.2)
Alkaline phosphatase (IU/L)	77.50 (63.50-89.25)	
Bacterial translation markers, median (IQR)		
sCD14	1708 (1478-2004)	
Zonulin	42.92 (38.52-47.16)	
I-FABP	749.7 (347.3-1256)	

*BMI*, Body mass index; *F*, female; *FCAP*, FibroScan-controlled attenuation parameter; *GERD*, gastroesophageal reflux disease; *I-FABP*, intestinal fatty acid binding protein; *IQR*, interquartile range; *kPa*, kilopascals; *M*, male; *Mc-QoL*, Mastocytosis Quality-of-Life; *sCD14*, serum CD14; *UP*, urticaria pigmentosa.

DNA extraction of stool samples was performed using Qiagen (formerly MoBio; Germany) PowerMag Microbiome DNA/RNA Isolation kit (cat# 27500-4-EP). The 16S rRNA gene was PCR amplified and sequenced on an Illumina (San Diego, Calif) Miseq as described^20^ (see this article’s Methods section in the Online Repository). Raw reads were denoised and trimmed for quality using the DADA2 pipeline to generate amplicon sequence variants (ASVs).^21^ Functional inference was performed using PICRUSt v2.3.0.^22^ Counts of ASVs and predicted functional genes were processed and statistically analyzed with R v4.4.2 (see this article’s Methods section in the Online Repository).

## Results

### Nutrition and symptoms

**Fig 1:**
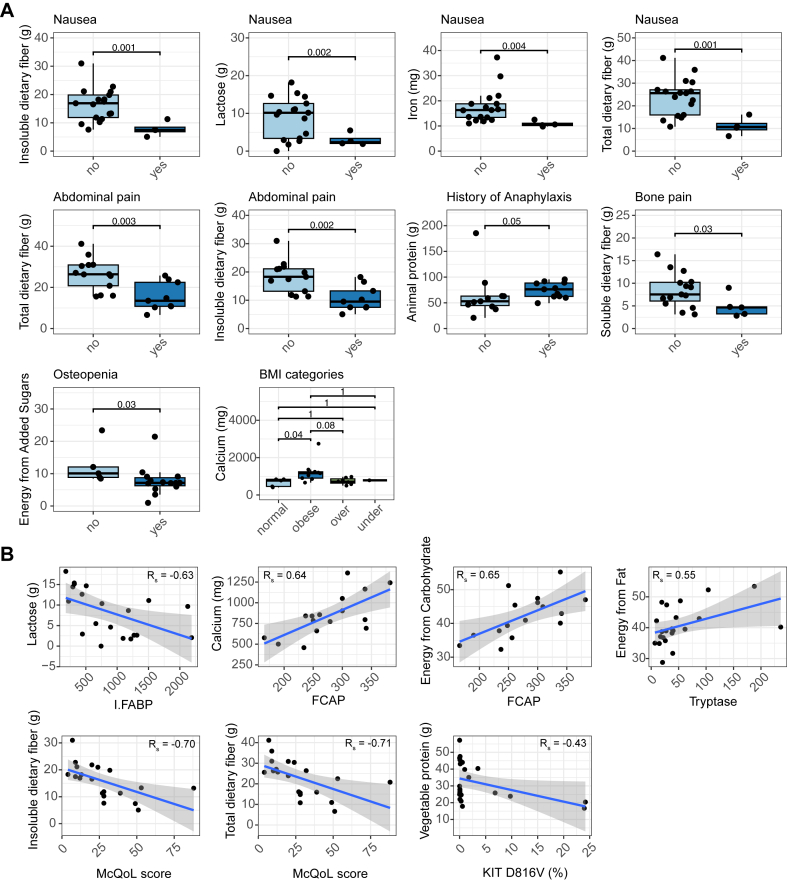
Relationship between SM and diet. **A,** Bone disease, GI, and allergic symptoms that are different based on dietary components. **B,** Associations between clinical markers and dietary components. *BMI*, Body mass index; *FCAP*, FibroScan-controlled attenuation parameter; *I-FABP*, intestinal fatty acid binding protein; *McQoL*, Mastocytosis Quality-of-Life.

Associations between diet and all reported symptoms, bacterial translocation markers, and markers for mast cell activation and inflammation reported for patients with SM were evaluated. Various aspects of nutrition were found to be related to bone disease, GI-related findings, anaphylaxis, body composition, quality of life, and markers of inflammation and mast cell activation (Fig 1). Patients who experienced bone pain consumed less soluble fiber (*P* = .03), and patients with osteopenia obtained less of their energy from added sugars than those who did not (*P* = .03; Fig 1, *A*). Abdominal pain (*P* = .003) and nausea (*P* = .001) were reported in patients who consumed less total dietary fiber, specifically insoluble dietary fiber (*P* = .002 and *P* = .001, respectively), and both negatively correlated with Mastocytosis Quality-of-Life scores (*R*_s_ = −0.71, *P* = .01, and *R*_s_ = −.70, *P* = .01, respectively; Fig 1, *A* and *B*). Nausea was also associated with a lower consumption of lactose (*P* = .002) and iron (*P* = .004), and lactose negatively correlated with intestinal fatty acid binding protein (*R*_s_ = 0.63, *P* = .02). Patients with a history of anaphylaxis consumed more protein from animals (*P* = .05, Fig 1, *A*). Consumption of calcium was positively correlated with FibroScan-controlled attenuation parameter (*R*_s_ = 0.64, *P* = .003) and was higher in patients with body mass index values in the category of obese (*P* = .04; Fig 1, *A* and *B*). FibroScan-controlled attenuation parameter was also positively correlated with consumption of energy from carbohydrates (*R*_s_ = 0.65, *P* = .009; Fig 1, *B*). Consumption of energy from fats positively correlated with serum tryptase (*R*_s_ = 0.55, *P* = .04), and protein from vegetables negatively correlated with KIT D816V (*R*_s_ = −0.43, *P* = .05; Fig 1, *B*). These data suggested that diet may influence the symptoms experienced by patients with SM, as well as manifestation of disease.

### Microbiome analysis

**Fig 2:**
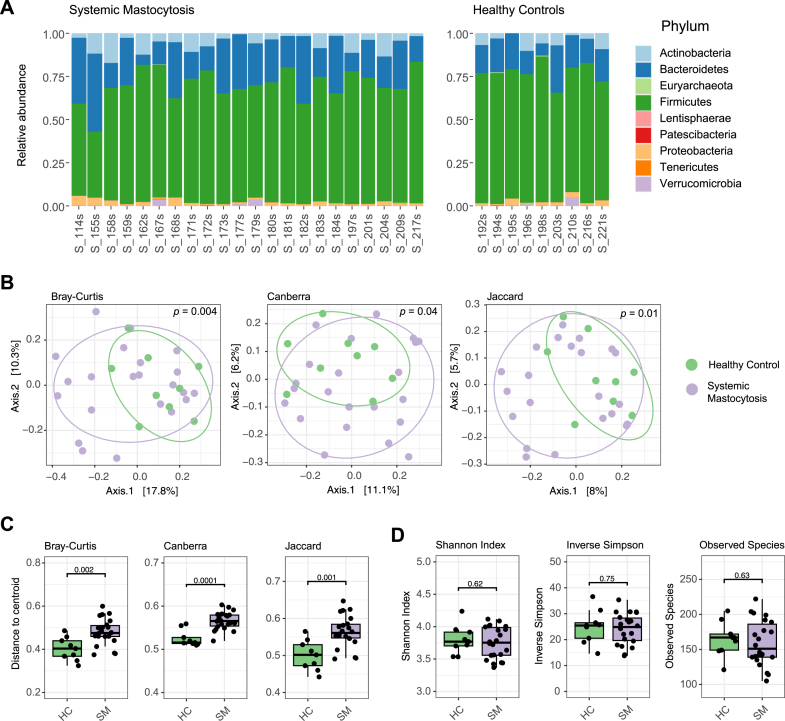
Microbial community structure, composition, and diversity between patients with SM and HCs, depicted as the relative abundance of phyla (**A**), PCoAs using Bray-Curtis dissimilarity, Canberra distance, and Jaccard index (**B**), beta dispersion using Bray-Curtis dissimilarity, Canberra distance, and Jaccard index compared with centroid (**C**), and measures of species richness and evenness (**D**).

The most abundant phyla across all patients were Firmicutes, followed by Bacteroidetes, Actinobacteria, Proteobacteria, Verrucomicrobia, Euryarchaeota, Tenericutes, Lentisphaerae, and Patescibacteria (Fig 2, *A*). Beta diversity (microbial community composition) was significantly different with consumption of vegetable protein (*P* = .005) and lactose (*P* = .05; see Fig E1 in this article’s Online Repository at www.jaci-global.org) but no other dietary components, sex, age, or race. When considering the confounding effect of vegetable protein and lactose, there were significant differences in microbial community composition between HCs and patients with SM (Bray-Curtis, *P* = .004; Canberra, *P* = .04; Jaccard, *P* = .01; Fig 2, *B*). There were also significant differences in beta dispersion (Bray-Curtis, *P* = .002; Canberra, *P* = .0001; Jaccard, *P* = .001), which evaluates the variation in microbial community composition and indicated that microbial composition was more variable among patients with SM (Fig 2, *C*). Alpha diversity metrics did not differ between patients with SM and HCs (*P* > .05; Fig 2, *D*) and did not correlate with KIT D816V or serum tryptase (*P* > .05; see Fig E2 in this article’s Online Repository at www.jaci-global.org). There were no differences in microbial composition or diversity based on symptoms, bacterial translocation markers, medications, or triggers within patients with SM. These observations suggested that changes in microbial community composition but not diversity were associated with SM.

**Fig 3:**
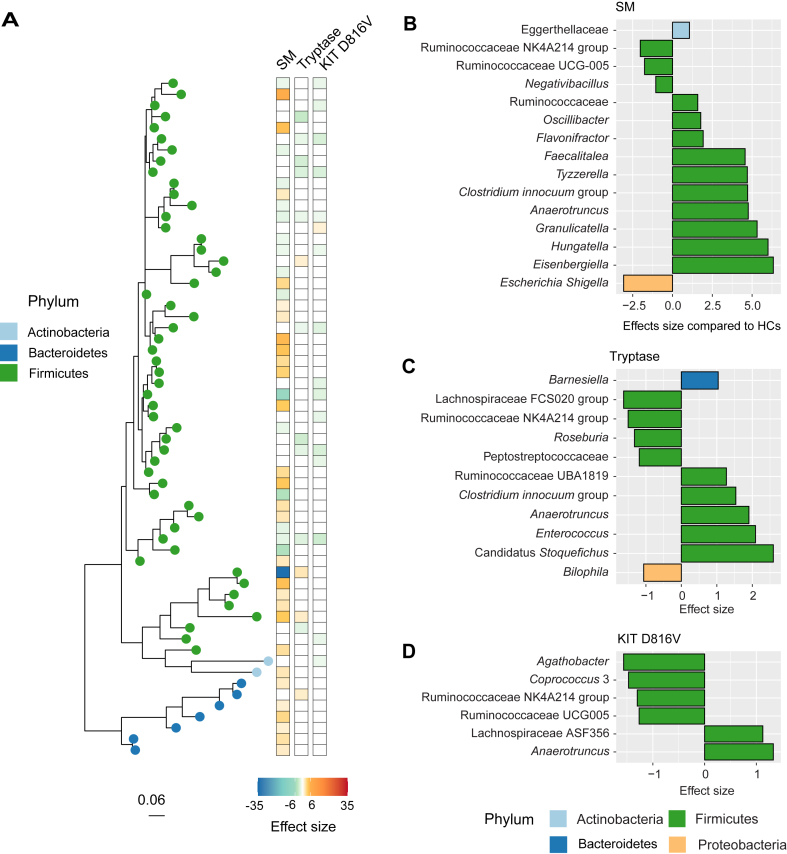
Taxa that were associated with SM, KIT D816V, and tryptase at the ASV level (**A**) and the genus level (**B-D**). Scale on the phylogenetic tree represents nucleotide substitutions per site. Effect sizes equal log_2_ fold change of the coefficient from MaAsLin2 and were significant after adjusting for multiple comparisons (*q* < 0.05).

Differentially abundant taxa were identified between those with SM and HCs (Fig 3, *A*; see Fig E3 in this article’s Online Repository at www.jaci-global.org). From the phylum Firmicutes, 20 ASVs were higher (adjusted *P* value [*q*] < .05, log_2_ fold > 1) and 22 ASVs were lower (*q* < 0.05, log_2_ fold < −1) in patients with SM. Six from the phylum Bacteroidetes and 1 from the phylum Actinobacteria were higher in patients with SM (Fig 3, *A*; Fig E3). Very few ASVs had species-level classification, and so differential abundance analysis was also done at genus level (Fig 3, *B*). From the phylum Firmicutes, *Negativibacillus*, Ruminococcaceae UCG005, and Ruminococcaceae NK4A214 group were lower in patients with SM. From the phylum Proteobacteria, *Escherichia-Shigella* was also lower in patients with SM. Genera from the phylum Firmicutes that were higher in patients with SM were *Eisenbergiella*, *Hungatella*, *Granulicatella*, *Anaerotruncus*, *Clostridium innocuum* group, *Tyzzerlla*, *Faecalitalea*, *Flavonifractor*, *Oscillibacter*, and an unclassified Ruminococcaceae, as well as the Actinobacterium Eggerthellaceae (Fig 3, *B*).

Taxa that were associated with serum tryptase and KIT D816V were also identified (*q* < 0.05, log_2_ fold > 1 or log_2_ fold < −1; Fig 3, *C* and *D*; see Figs E4 and E5 in this article’s Online Repository at www.jaci-global.org). *Anaerotruncus*, which was also higher in patients with SM, had a positive relationship with serum tryptase and KIT D816V. Ruminococcaceae NK4A214 had a negative relationship with KIT D816V and serum tryptase, in addition to being lower in patients with SM. However, there were unique signatures associated with biomarkers. *Bilophila*, a Proteobacteria, and Peptostreptococcaceae, Lachnospiraceae FCS020 group, and *Roseburia*, from the phylum Firmicutes, had a negative relationship with serum tryptase. Bacteroidetes genera *Barnesiella* and the Firmicutes genera Ruminococcaceae UBA1819, *Enterococcus*, *Clostridium innocuum* group (also higher in patients with SM), and Candidatus *Stoquefichus* had a positive relationship with serum tryptase concentrations (Fig 3, *C*). The Firmicutes genera *Agathobacter*, Ruminococcaceae UCG005 (also lower in patients with SM), and *Coprococcus* 3 had a negative relationship with KIT D816V, and the Firmicute Lachnospiraceae ASF356 had a positive relationship with KIT D816V (Fig 3, *D*). These data support the observations that there was some coherence in taxa associated with SM and markers of mast cell activation, but there were also unique microbial signatures for each.

**Fig 4:**
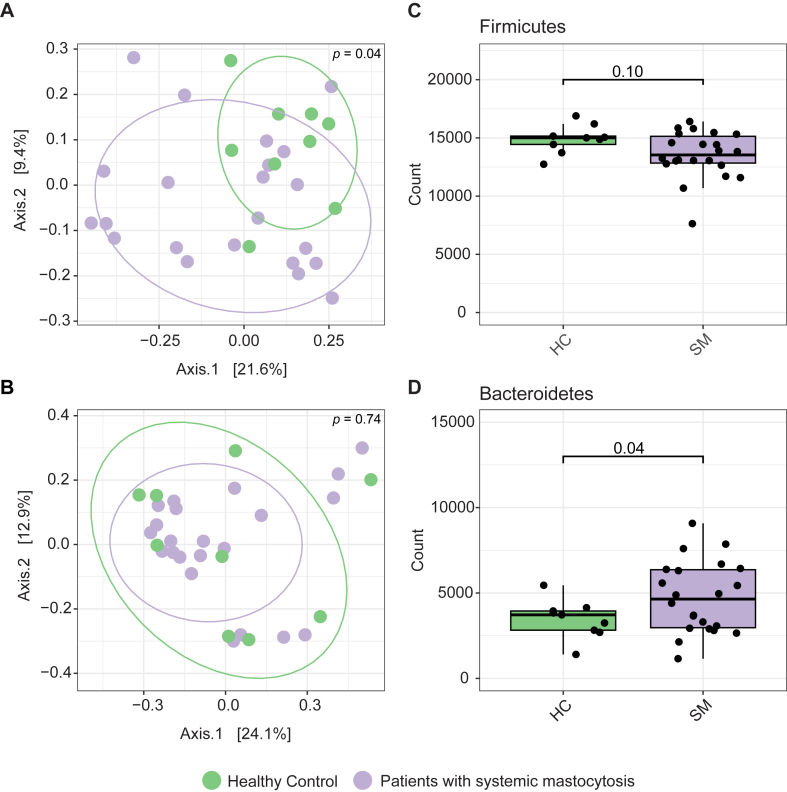
Microbial community composition of Firmicutes (**A**) and Bacteroidetes (**B**) depicted as PCoAs using Bray-Curtis dissimilarity and differences in the abundance of Firmicutes (**C**) and Bacteroidetes (**D**) between HCs and patients with SM.

Differentially abundant taxa between patients with SM and HCs were almost entirely Firmicutes and Bacteroidetes. The microbial community composition of Firmicutes alone was different between patients with SM and HCs (*P* = .04, Fig 4, *A*) and not different when Bacteroidetes was examined alone (*P* = .74, Fig 4, *B*). Abundances of Firmicutes did not differ between the 2 groups (*P* = .10; Fig 4, *C*); however, Bacteroidetes were significantly higher in patients with SM compared with HCs (*P* = .04; Fig 4, *D*). This did not translate to differences in Firmicutes to Bacteroidetes ratio (*P* = .145). This suggested that dysbiosis in patients with SM was defined by the composition of Firmicutes and increased abundance of Bacteroidetes. Although these significant results were close to the threshold, they aligned with differential abundance analysis, indicating Bacteroidetes were enriched in patients with SM compared with HCs (Fig 3, *A*) and changes in Firmicutes composition were drivers of the differences (Fig 3, *B-D*).

### Functional inference

PICRUSt2 was used to predict functional genes in the microbial community of patients with SM and HCs. Gene composition was associated with consumption of energy from fat (*P* = .03) and carbohydrates (*P* = .04) as well as total protein (*P* = .008) in patients with SM (see Table E2 in this article’s Online Repository at www.jaci-global.org) but not in HCs (*P* > .05; Table E2). This indicated that diet could be more influential in functional composition of patients with SM compared with HCs.

**Fig 5:**
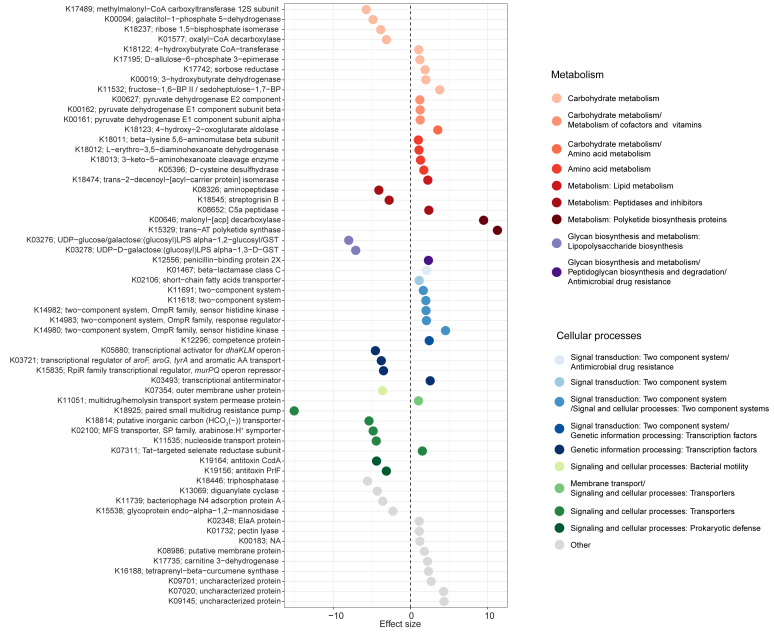
Differentially abundant genes predicted using PICRUSt2 from 16S rRNA sequences. All genes are significant after adjusting for multiple comparisons (*q* < 0.05). Effect sizes equal log_2_ fold change of the coefficient from MaAsLin2 and were significant after adjusting for multiple comparisons (*q* < 0.05).

There were 60 differentially abundant predicted genes observed between HCs and patients with SM (*q* < 0.05, log_2_fold > 1 or log_2_fold < −1, Fig 5). Several encoded proteins for carbohydrate metabolism. Genes encoding pyruvate dehydrogenase E1 components alpha (K00161) and beta (K00162), pyruvate dehydrogenase E2 component (K00627), and fructose-1,6-phosphatase II (K11532) were higher in patients with SM. Several genes encoded proteins for short-chain fatty acid (SCFA) metabolism, including 3-hydroxybutyrate dehydrogenase (K00019) and 4-hydroxybutryate CoA-transferase (K18122), which were higher in patients with SM, and methyl malonyl-CoA carboxyltransferase 12S subunit (K17489), which was lower. Patients with SM were enriched in genes involved in pathways for lysine fermentation, including 3-keto-5-aminohexanoate cleavage enzyme (K18013), l-erythro-3,5-diaminohexanoate dehydrogenase (K18012), and beta-lysine 5,6-aminomutase beta subunit (K18011). An SCFA transporter (K02106) was also higher in patients with SM. This suggested that dysbiosis in patients with SM likely translates into differences in carbohydrate and SCFA metabolism.

Some differentially abundant genes also encoded peptidases and proteins involved in glycan metabolism and polyketide synthesis (Fig 5). The gene encoding the peptidase Streptogrysin B (K18545) was lower in patients with SM, and a C5a peptidase (K08652) was higher in patients with SM. Two polyketide synthesis genes, trans-AT polyketide synthase (K15329) and malonyl-[acp] decarboxylase (K00646), were higher in patients with SM. Glycan biosynthesis and metabolism in the context of lipopolysaccharide biosynthesis were lower in patients with SM (K03278 and K03276); however, penicillin-binding protein 2X (K12556), involved in peptidoglycan synthesis and antimicrobial resistance, was higher.

Other differentially abundant genes were involved in signal transduction, cell signaling, genetic information processing, and transport (Fig 5). Patients with SM were enriched in genes encoding 2 component systems, including beta lactamase class C (K01467), OmpR family sensor histidine kinases (K14982 and K14980), and response regulators (K14983). Genes for transcriptional regulation of amino acids, lipids, and cell wall sugars were lower in patients with SM, including one for aromatic amino acid transport (K03721), the *dhaKLM* operon (K05880), and the *murPQ* operon (K15835), respectively. Most genes encoding transporters for multidrug resistance (K18925), inorganic carbon (K18814), nucleosides (K11535), and a major facilitator transporter (K02100) were also lower in patients with SM. In addition, genes encoding CcdA (K19164) and PrlF (K19156), antitoxins of type II toxin-antitoxin systems, were lower in patients with SM. These genes involved in transcriptional regulation, transport, virulence, and microbial defense may thus impact microbial metabolism in patients with SM.

## Discussion

We explored the role of the gut microbiome in patients with SM and observed dysbiosis, defined by changes in composition of Firmicutes and an increased abundance of Bacteroidetes (Figs 2 and 4), and unique microbial taxa associated with markers of inflammation and mast cell expansion and activation (Fig 3). Predicted genes in the gut microbiome indicated that there are likely to be differences in microbial metabolism in patients with SM, specifically carbohydrate and amino acid metabolism, SCFAs, virulence factors, and bacterial defense (Fig 5). In addition, diet was associated with microbial community composition and may also influence occurrences of symptoms and markers for mast cell activation and inflammation (Fig 1).

### Diet influences the microbiome and clinical manifestations of patients with SM

Diet likely impacts mast cell function.^23^^,^^24^ In this study, there were differences in clinical manifestations of SM, including quality of life, associated with a patient’s diet (Fig 1). Generally, these results may suggest that a diet lower in animal protein and energy from fat, higher in protein from vegetables, fiber, lactose, and minerals, such as iron, may improve GI and bone symptoms as well as reduce episodes of anaphylaxis. However, this was an association study with no matched control group, and some *P* values were borderline significant. Thus, more work needs to be done to verify these possibilities and should be cautiously interpreted.

The association of diet and clinical manifestations observed here may be reflective of how patients with SM already manage their symptoms. For example, some patients with SM who experience irritable bowel syndrome–like symptoms claim that avoiding fiber and dairy may diminish flare ups.^25^ This may explain why a decreased consumption of fiber was associated with nausea, abdominal pain, and bone pain, and an increase in fiber consumption was associated with increased reported quality of life (Fig 1). Also, previous studies have reported certain foods to be linked to allergies and anaphylaxis in patients with mast cell diseases,^26^ and certain dietary compounds, including dietary fiber, have been shown to influence mast cell activation.^23^^,^^26^ Thus, some of these associations may be meaningful but remain only hypothesis generating. Further work to investigate different sources of these dietary components could be helpful in identifying more specific triggers in patients with SM.

Predicted functional gene composition in patients with SM was also uniquely influenced by diet (Table E2), suggesting that microbial metabolism in the gut of patients with SM is more susceptible to changes compared with HCs. These findings support the notion that diet may contribute to gut dysbiosis in patients with SM.

### SM is associated with gut microbiome dysbiosis

SM and mast cell burden and clonality, as reflected by serum levels of tryptase and frequency of the KIT D816V, were associated with altered microbial community composition (Figs 2 and 3). A previous study examining the gut microbiome in patients with SM also showed differences in microbial community composition and taxa that correlated with serum tryptase, in addition to lower diversity using Shannon Index.^27^ We, however, did not observe differences in alpha diversity metrics, including Shannon Index (Fig 2, *D*). This could be due to several factors including the geographic location of cohorts, severity of symptoms, diet, or management of disease, all of which could well influence the gut microbiome.^28^ Despite these possible confounders, our studies agree that there is dysbiosis associated with SM. We also found that there was more variability in microbial composition in patients with SM (Fig 2, *C*), which may be a function of instability of the gut microbiome or depict differences driven by an individual’s combination of medications or clinical manifestations. We additionally identified unique microbial signatures associated with SM, KIT D816V, and serum tryptase, indicating that each relate to different responses from the gut microbiome.

The composition of Firmicutes and an increased abundance of Bacteroidetes defined differences in the gut microbiome of SM and associated with KIT D816V and serum tryptase (Fig 4). Firmicutes and Bacteroidetes are the most abundant phyla in the gut microbiome, and the relationship between these 2 groups has been linked to several diseases, such as bowel disorders and obesity.^29^ Some Firmicutes have also been shown to interact with mast cells. For example, pathogenic Firmicutes can elicit a response from mast cells leading to degranulation, including *Streptococcus* and *Listeria*,^30^ whereas others, such as *Enterococcus faecalis* and probiotic Lactobacilli, can attenuate mast cell degranulation.^30^ Both Firmicutes and Bacteroidetes are also producers of metabolites that regulate the immune system and are important in gut barrier integrity, such as SCFAs, amino acids, polyamines, and vitamins.^31^

Some opportunistic pathogens were associated with SM and markers, such as *Clostridium innocuum*, which was higher in SM and increased with serum tryptase, and *Enterococcus*, which was also higher with increased serum tryptase (Fig 3). These taxa are considered emerging pathogens, causing nosocomial infections and increasingly acquiring antibiotic resistance, and thus could contribute to dysbiosis and inflammation in the gut.^32^^,^^33^ Conversely, *Escherichia-Shigella* complex was lower in SM, likely representing *Escherichia,* because no patients had Shigellosis and *Shigella* has a low infectious dose. Although *Escherichia* includes both pathogenic strains and opportunistic pathogens, many *Escherichia* (ie, *Escherichia coli*) are part of normal and healthy flora.^34^ In fact, previous work showed a reduction in colony-forming units of nonpathogenic *E coli* in patients with SM^27^ (Fig 3, *B*), which would align with our observation at the molecular level. *E coli* can also inhibit degranulation of mast cells,^35^ suggesting a possible compelling interaction between the microbiome and SM. Several of the genera higher in SM have been linked to disease, including *Anaerotruncus*,^36^^,^^37^ *Tyzzerella*,^38^ *Oscillibacter*,^39^ *Granulicatella*,^40^ *Flavonifractor*,^41^ and *Eisenbergiella*^42^ (Fig 3). However, these are also commensals and may have protective effects or attenuate inflammation.^40^^,^^41^^,^^43^, ^44^, ^45^ The same is true for other members of the Ruminococcaceae and Lachnospiraceae families,^46^^,^^47^ including *Roseburia*,^48^^,^^49^ *Coprococcus*,^50^ and *Hungatella*,^51^ as well as *Negativibacillus*,^52^ which explains why some are positively and negatively associated with SM and mast cell markers. Although we can now hypothesize a role for some of these taxa in SM, gene predictions from the 16S rRNA gene were performed to investigate metabolic differences in the gut microbiome that may arise from compositional changes and influence patients with SM.

### Dysbiosis in SM translates to altered microbial metabolism

Differential predicted gene abundances suggest that dysbiosis observed in the gut microbiome in SM is translated to altered metabolic potential (Fig 5). Broadly, these functional genes suggest that there are differences in the metabolic pathways for carbohydrate, amino acid, glycan, lipid, vitamin, and cofactor metabolism. In addition, there were higher abundances of genes encoding polyketide synthesis proteins in patients with SM and differentially abundant genes encoding for transporters.

Activity of these genes could lead to different metabolites produced by the gut microbiome that could interact with the host physiology, such as SCFAs. SCFAs, such as butyrate, propionate, and acetate, are produced by the gut microbiome, and their production is influenced by diet and microbial composition and diversity.^53^ They are important regulators of gut integrity as well as the immune system, including mast cell function.^18^ In this study, patients with SM had higher abundances of genes involved in butyrate metabolism, lysine fermentation (which leads to butyrate production), and an SCFA transporter, as well as many carbon cycling genes that intersect with SCFA production pathways. HCs were enriched in a different set of genes encoding proteins involved in metabolism of carbohydrates and SCFAs, such as propionate and formate, as well as aromatic amino acid metabolism, which are precursors to metabolites that regulate immune and metabolic responses of the host. Thus, there were likely differences in mechanisms for production of SCFAs and other metabolites that interact with the immune system in patients with SM. It has been reported that patients with SM have decreased gut barrier integrity,^54^ and this could indicate that dysbiosis leads to altered production of these metabolites. This supports the need for further investigation of the role of SCFAs and other microbial metabolites in the clinical manifestations of SM.

Other predicted genes enriched in patients with SM indicate that they carried more virulent microorganisms, coinciding with the identification of more opportunistic genera. Genes encoding virulence factors, such as antimicrobial resistance, competence, hemolysin transport, and degradation of complement protein C5, were enriched in patients with SM. These observations support the concept that the microbiome of patients with SM has the capacity to be more pathogenic by evading antibiotics, incorporating exogenous DNA, producing toxins, and evading the immune system. Patients with SM were also predicted to be enriched in 2 component systems, important signaling mechanisms that respond to extracellular cues allowing for adaptation to changing environments.^55^ Indeed, some enriched in SM are involved in cell response to extracellular stress, pathogenesis, and antibiotics.^56^ Fewer genes for bacteriophage resistance and antitoxins for type II toxin-antitoxin systems were predicted as well, indicating that the microbiome was less equipped to defend against phage infection and microbial toxins that lead to cell death. Therefore, patients with SM may have a gut microbiome that is more susceptible to dysbiosis from microbial antagonists.

In this study, we observed changes in the composition of the gut microbiome in patients with SM, proportion of mast cells with the KIT D816V mutation, and increased mast cell activation. We further show that this likely leads to altered microbial metabolism, indicating that there is dysbiosis associated with SM. Diet also influenced microbial composition and metabolism as well as clinical manifestations of disease in patients with SM. Although the sample size for this study was small, these results offer opportunities to generate new hypotheses related to the potential interactions of the gut microbiome and SM. Further investigation with a larger sample size to enable consideration of management of disease and other manifestations and variants of SM will be useful to elucidate the clinical significance of these findings.

## Disclosure Statement

This research was funded by the Division of Intramural Research, National Institute of Allergy and Infectious Diseases (NIAID), National Institutes of Health (NIH). Funding for L.E.K. was in part with Federal funds from the NIAID, Department of Health and Human Services under Bioinformatics and Computational Bioscience Branch (BCBB) Support Services Contract HHSN316201300006W/75N93022F00001 to Guidehouse 10.13039/100031019Digital.

Disclosure of potential conflict of interest: All the authors declare that they have no relevant conflicts of interest.
