# Supplementary material for: Evidence for dysbiosis in the gut microbiome of patients with systemic mastocytosis
Source: J Allergy Clin Immunol Glob. 2025 Oct 9;5(1):100578. doi: 10.1016/j.jacig.2025.100578 (PMC12826983; doi:10.1016/j.jacig.2025.100578)
Supplement: Supplemental Table and Figures [file mmc1.docx]

**Supplemental Table S2 and Figures S1-S5**

**Title: Evidence for dysbiosis in the gut microbiome of patients with systemic mastocytosis**

Lauren E. Krausfeldt, PhD^1^, Vivian Cao, MS^2,3^, Richard Rodrigues, PhD^4,5^, Wendy A. Henderson, PhD^6,7^, Robin Eisch, RN^2^, Linda M. Scott, LNP^2^, Dean D. Metcalfe, MD, MS^2^, Hirsh D. Komarow, MD^2*^

^1^Bioinformatics & Computational Biosciences Branch, Office of Cyber Infrastructure and Computational Biology, National Institute of Allergy and Infectious Diseases, National Institutes of Health, Bethesda, Maryland

^2^Mast Cell Biology Section, Laboratory of Allergic Diseases, National Institute of Allergy and Infectious Diseases, National Institutes of Health, Bethesda, Maryland

^3^University of Pittsburgh School of Medicine

^4^Microbiome and Genetics Core, Laboratory of Integrative Cancer Immunology, Center for Cancer Research, National Cancer Institute, Bethesda, MD 20852, USA

^5^Basic Science Program, Frederick National Laboratory for Cancer Research, Frederick, MD 21701, USA

^6^Digestive Disorders Unit, National Institute of Nursing, National Institutes of Health, Bethesda, Maryland

^7^Department of Biobehavioral Health Sciences, University of Pennsylvania, Philadelphia, PA 19104-4217

*Corresponding Author:

Hirsh D. Komarow, M.D.

Associate Research Physician

Laboratory of Allergic Diseases, NIAID, NIH

Building 10, Room 6D44A

10 Center Drive,

Bethesda, MD 20892-1960

301 594-2197

301-402-4271 FAX

[komarowh@mail.nih.gov](mailto:komarowh@mail.nih.gov)

Table S2. Differences in functional composition (PERMANOVA) in patients with systemic mastocytosis (SM) and healthy controls (HCs) based on dietary intake. Bolded *p* values are significant.

|  | **Patients with SM** | |  | **HCs** | |
| --- | --- | --- | --- | --- | --- |
|  | R^2^ | *p* |  | R^2^ | *p* |
|  |  |  |  |  |  |
| Energy from added sugars | 0.08949 | 0.098 |  | 0.165 | 0.214 |
| Energy from carbohydrate | **0.11766** | **0.035** |  | 0.0751 | 0.866 |
| Energy from fat | **0.11625** | **0.034** |  | 0.10396 | 0.607 |
| Energy from protein (g) | 0.06214 | 0.213 |  | 0.08898 | 0.78 |
| Insoluble dietary fiber (g) | 0.06595 | 0.167 |  | 0.19531 | 0.09 |
| Soluble dietary fiber (g) | 0.05442 | 0.303 |  | 0.13508 | 0.34 |
| Total dietary fiber (g) | 0.06586 | 0.213 |  | 0.20713 | 0.06 |
| Total protein (g) | **0.1759** | **0.008** |  | 0.14739 | 0.26 |
| Vegetable protein (g) | 0.08219 | 0.093 |  | 0.17713 | 0.114 |
| Animal protein (g) | 0.09372 | 0.091 |  | 0.12829 | 0.431 |
| Lactose (g) | 0.04105 | 0.466 |  | 0.07821 | 0.804 |
| Calcium (mg) | 0.02315 | 0.813 |  | 0.07114 | 0.929 |
| Iron (mg) | 0.03313 | 0.613 |  | 0.13347 | 0.365 |
| Zinc (mg) | 0.02916 | 0.706 |  | 0.09122 | 0.748 |
|  |  |  |  |  |  |

Figure S1. PCoA depicting relationships between samples based on Bray Curtis dissimilarity colored by tryptase (ng/mL), KIT D816V (% mast cells), consumption of vegetable protein (g), and consumption of lactose (g).

Figure S2. Spearman correlations between KIT D816V and tryptase to alpha diversity metrics. “X” represents no significance with p value > 0.05.

Figure S3. Differentially abundant ASVs between HCs and patients with SM. Colors represent phylum. ASVs are labeled with their highest resolution taxonomic classification. The effect size is equal to log_2_ fold change of the coefficient produced by MaAsLin2. All taxa are significant with a *q* of < 0.05.

Figure S4. Differentially abundant ASVs with tryptase levels. Colors represent the phylum that the ASV belongs. ASVs are labeled with their highest resolution taxonomic classification. Effect size equals the log_2_ fold change of the coefficient produced by MaAsLin2. All taxa are significant with a *q* of < 0.05.

Figure S5. Differentially abundant ASVs with KIT D816V. Colors represent the phylum that the ASV belongs. ASVs are labeled with their highest resolution taxonomic classification. Effect size equals the log_2_ fold change of the coefficient produced by MaAsLin2. All taxa are significant with a *q* of < 0.05.
