# Supplementary material for: Evidence for dysbiosis in the gut microbiome of patients with systemic mastocytosis
Source: J Allergy Clin Immunol Glob. 2025 Oct 9;5(1):100578. doi: 10.1016/j.jacig.2025.100578 (PMC12826983; doi:10.1016/j.jacig.2025.100578)
Supplement: Supplemental_Methods [file mmc2.docx]

**Supplemental Methods**

**Title: Evidence for dysbiosis in the gut microbiome of patients with systemic mastocytosis**

Lauren E. Krausfeldt, PhD^1^, Vivian Cao, MS^2,3^, Richard Rodrigues, PhD^4,5^, Wendy A. Henderson, PhD^6,7^, Robin Eisch, RN^2^, Linda M. Scott, LNP^2^, Dean D. Metcalfe, MD, MS^2^, Hirsh D. Komarow, MD^2*^

^1^Bioinformatics & Computational Biosciences Branch, Office of Cyber Infrastructure and Computational Biology, National Institute of Allergy and Infectious Diseases, National Institutes of Health, Bethesda, Maryland

^2^Mast Cell Biology Section, Laboratory of Allergic Diseases, National Institute of Allergy and Infectious Diseases, National Institutes of Health, Bethesda, Maryland

^3^University of Pittsburgh School of Medicine

^4^Microbiome and Genetics Core, Laboratory of Integrative Cancer Immunology, Center for Cancer Research, National Cancer Institute, Bethesda, MD 20852, USA

^5^Basic Science Program, Frederick National Laboratory for Cancer Research, Frederick, MD 21701, USA

^6^Digestive Disorders Unit, National Institute of Nursing, National Institutes of Health, Bethesda, Maryland

^7^Department of Biobehavioral Health Sciences, University of Pennsylvania, Philadelphia, PA 19104-4217

*Corresponding Author:

Hirsh D. Komarow, M.D.

Associate Research Physician

Laboratory of Allergic Diseases, NIAID, NIH

Building 10, Room 6D44A

10 Center Drive,

Bethesda, MD 20892-1960

301 594-2197

301-402-4271 FAX

komarowh@mail.nih.gov

**Table of contents**

Assessment of clinical and molecular correlates (page 2)

16S rRNA gene amplicon library preparation and sequencing (page 2)

Bioinformatic processing and statistics (page 3)

Dietary assessment (page 5)

*Assessment of clinical and molecular correlates*

Symptoms were reported if they were chronic and recurrent (Table S1). GI symptoms assessed included chronic nausea, GERD, recurrent abdominal pain, and occurrence of diarrhea. Bone health was assessed by findings of chronic bone pain, osteoporosis, and osteopenia. Allergic symptoms included history of anaphylaxis and flushing. Neurological symptoms were assessed with brain fog, headaches, and depression. Quality of life was assessed with the mastocytosis quality of life (MC-QoL) score, where a lower score suggested better QoL (1). Markers of mast cell activation and inflammation measured in all subjects and included tryptase levels, FibroScan controlled attenuation parameter (FCAP) to measure liver scarring, and kPa to measure of liver elasticity. The presence of urticaria pigmentosa (UP) was noted. In patients with SM, frequency of the KIT D816V mutation was also detected by allele-specific quantitative PCR (qPCR) both in peripheral blood and bone marrow. Bacterial translocation markers soluble CD14 (sCD14), zonulin, and intestinal fatty acid binding protein (I-FABP) were measured in serum of patients with SM. Medications and triggers were also recorded (Table 1, Table S1). All patients were born and raised in the United States.

*16S rRNA gene amplicon library preparation and sequencing*

A first PCR amplification step was performed using primers specific to the V4 region (515F-806R) of the 16S rRNA gene and Phusion High Fidelity PCR Mastermix. The PCR was performed with the following conditions: 1 cycle at 98°C for 30 seconds, 10 cycles at 98°C for 10 seconds, 60°C for 30 seconds (with 1 degree decrease/cycle), 70°C for 30 seconds, 7 cycles of 98°C for 30 seconds, 50°C for 30 seconds, 72°C for 30 seconds, and a final elongation step at 72°C for 7 minutes. The PCR product was purified with AMPure XP beads (Beckman Coulter, Brea, CA,Cat# A63881), and a second PCR was performed for barcode ligation with Illumina (San Diego, CA) Nextera XT V2 kit with 2X Phusion High Fidelity Mastermix. This PCR was performed with the following conditions: 1 cycle at 98°C for 30 seconds, 8 cycles at 98°C for 10 seconds, 55°C for 30 seconds, 70°C for 30 seconds, and a final elongation step at 72°C for 7 minutes. Final PCR product was purified AMPure Beads and concentration was determined with KAPA Library quantification kit (cat#KK4873, Kapa Biosystems, Wilmington, MA) on a Quantstudio 6 Flex (ThermoFisher, Waltham, MA). The libraries were pooled at the same concentration. All steps were automated by using epMotion liquid handler 5073 and 5075 (Eppendorf, Germany). Libraries were sequenced on an Illumina Miseq with 10% PhiX spike in with 2 x 261 cycles for 250 bp paired end reads (2, 3).

*Bioinformatic processing and statistics*

Raw counts from DADA2(4) and PICRUSt2(5) were filtered using the package metagMisc in R v 4.4.2 with a prevalence threshold of 0.1 and abundance threshold of 10 and rarefied at 20,000 reads to account for differences in read depth and counts of predicted genes from PICRUSt2 were rarefied to 5,500,000. Alpha and beta diversity were assessed with the R packages *vegan* and *phyloseq*.(6, 7) (8) and functional gene composition was examined using Principal Coordinates Analysis (PCoA), Permutational Multivariate Analysis of Variance (PERMANOVA), and beta dispersion with Bray Curtis dissimilarity, Canberra distance, and Jaccard index. Other variables, related to dietary components, sex, race, age, and the presence of UP were evaluated individually for associations with microbial composition as well as included in the model to consider their confounding or interactive effect. They were not confounders and had no interactive effect with disease (SM), the main effect. Alpha diversity was evaluated using Shannon Index, Inverse Simpson, and Observed Species, and functional diversity was evaluated using observed species. Alpha diversity, functional diversity, clinical symptoms, and nutritional markers were compared using t.test or Wilcoxon test between two groups while ANOVA and Tukey’s test or Kruskal Wallis and Dunn’s test for more than two groups based on results of Shapiro’s test for normality. Spearman correlations using R package *rcorr* were used to identify relationships between continuous variables and a linear regression model from the R packages *stats* was used to identify confounding effects of diet on alpha diversity. Medians and interquartile ranges (IQR) were calculated in GraphPad Prism (San Diego, CA). Differential abundances of ASVs and predicted functional genes between groups or corresponding to symptoms were determined using *MaAsLin2*(9) with negative binomial model and Benjamin-Hochberg Procedure to correct for multiple comparisons to produce adjusted p values (q value). For genus level analysis, ASVs were summed by genus and analyzed the same way. A phylogenic tree with differentially abundant ASVs was made with FastTree(10) after aligning ASVs with MAFFT.(11) Coefficients produced by MaAsLin2 were converted to log_2_ fold change to represent effect size. ASVs, genera, and functional genes were considered significant with a q value < 0.05, log2fold change of < -1 or >1, and where the number of samples with non-zero values were > 9. Confounding dietary variables identified with PERMANOVA were also incorporated into differential abundance analysis. Functions of differentially abundant genes was identified using descriptions from Kyoto Encyclopedia of Genes and Genomes (KEGG) and KEGG Orthology (KO) IDs. Sequencing reads are available at NCBI’s Sequence Read Archive under Bioproject PRJNA1258241.

*Dietary assessment*

For dietary assessment, participants recorded their diet for three days prior to sample collection. The final dietary assessment was converted to predicted consumption of calcium (mg), iron (mg), zinc (mg), potassium (mg), percentage energy as fat, percentage energy as carbohydrate, percentage energy as added sugars, total protein (g), animal protein (g), vegetable protein (g), lactose (g), total dietary fiber (g), soluble fiber (g), insoluble fiber (g), and lysine (g). Participants were instructed to keep food records and in the format as described and shown below.

# Keeping Food Records

We need to assess your usual food and beverage intake. For this reason, we are asking you to write down everything you eat and drink for 3 days.

***When should the food records be kept?*** Write down all foods and beverages you consume for 3 consecutive days prior to your NIH visit. Eat normally on these days. Don’t change your food choices, methods of food preparation, or where you eat just because you are recording your intake. There is no right or wrong way to eat for this evaluation.

***How and what should be recorded?*** Use a new form each day. It is best to record what you eat *immediately* after each meal and snack.

You should write down:

- the time you begin eating each meal and snack.
- all foods and beverages you consume, except for plain water. Don’t forget to record candy, mints, etc.
- all condiments (such as ketchup, margarine, mayonnaise, salad dressing, sauces, gravy, sugar, etc). You *do not* need to record salt, pepper, herbs and spices.
- all medicines, vitamins, minerals, and/or other supplements taken.

For each food and beverage that you list, include an amount and a description. To guide you, refer to the sample food record and the hints for recording amounts and description information on the next few pages.

***What do I do with the forms after they are filled out?*** Bring the completed forms with you to your NIH visit. A dietitian will review the food records with you in case any items need clarification.

**Hints for Completing the *Amount* Column**

A. Measure foods *after* preparation and cooking is completed.

B. Measurements can be listed in 4 ways:

1. The number of items.

*Examples:* saltines 6

white bread 2 SL

grapes 12

ketchup 2 packets

2. In household measures, using standard level measuring cups and spoons.

*Examples:* applesauce 1/3 CP

popcorn 3 cups

jelly 1 1/2 TB

2% milk 1.5 CP

(Do not use non-standard measures like “handful” or “serving”)

3. By weight or by volume, as listed on a package (or by using a kitchen scale if you have one).

*Examples:* gingerale 12 FL OZ

yogurt 4.5 oz

Almond Joy 49 gm

roast beef 2 ½ oz

pretzels 1/3 of 6.5 oz bag

4. By dimensions, using a ruler.

*Examples:* pancake 5” diameter

meatball 1 ¼” diameter

lasagna 3 ½” x 4” x 1½” rectangle

pizza 1/8 of 14” diameter [or sketch it w/ dimensions]

C. Remember not all food that is served is eaten, and at other times, you may go back for seconds. You may need to adjust portion sizes to reflect the amount you actually ate.

**Hints for Completing the *Description* Column**

A. Describe foods completely.

*Examples:*

*sirloin* steak, *fat partially trimmed*

*80% lean* ground beef (or 80/20 ground beef)

chicken drumstick, *skin removed before cooking*

*reduced fat* or *2%* milk

*baked* potato, *skin eaten*

*carrot* cake *with cream cheese frosting*

B. Include brand names whenever possible. Also include terms like *calcium-fortified, light*, and *reduced calorie* if listed on the label.

*Examples*:

*Country Crock Light spread* tub margarine

*Oreo* *reduced-fat* cookies

*Miller Lite* draft beer

*Hellmann’s fat-free cholesterol-free* mayonnaise

*Dannon lowfat* fruited yogurt

sliced peaches in *light* syrup

C. Include information about preparation and cooking methods.

*Examples:*

skinless chicken breast *floured and pan-fried in corn oil*

canned corn *tub margarine and sugar added*

Campbell’s tomato soup *made with water*

mac and cheese  *from mix, made with whole milk and Parkay stick margarine*

D. For mixed dishes and recipe items, you only need to list major ingredients. You do not need to write down the entire recipe.

*Examples:*

potato salad *made with potatoes, eggs, regular mayo*

chocolate chip cookies *homemade with real butter, walnuts added*

meatloaf  *made with 85/15 ground beef, oatmeal, ketchup, egg*

E. For fast food items from major chains, you only need to name the item. No description is necessary, unless you “special order” an item. Also, note if all of it wasn’t eaten.

*Examples:*

*Biggie Fries (Wendy’s)*

*Quarter Pounder with Cheese (McDonald’s) - didn’t eat pickles*

*Meat-lovers Pan Pizza (Pizza Hut)*

F. Remember to record any additions made at the table, such as margarine, sugar, ketchup, mustard, sauces, mayonnaise. List them separately, and include amounts.





References

1. Siebenhaar F, von Tschirnhaus E, Hartmann K, Rabenhorst A, Staubach P, Peveling-Oberhag A, et al. Development and validation of the mastocytosis quality of life questionnaire: MC-QoL. Allergy. 2016;71(6):869-77.

2. Rosshart SP, Herz J, Vassallo BG, Hunter A, Wall MK, Badger JH, et al. Laboratory mice born to wild mice have natural microbiota and model human immune responses. Science. 2019;365(6452).

3. Ma C, Han M, Heinrich B, Fu Q, Zhang Q, Sandhu M, et al. Gut microbiome-mediated bile acid metabolism regulates liver cancer via NKT cells. Science. 2018;360(6391).

4. Callahan BJ, McMurdie PJ, Rosen MJ, Han AW, Johnson AJ, Holmes SP. DADA2: High-resolution sample inference from Illumina amplicon data. Nat Methods. 2016;13(7):581-3.

5. Douglas GM, Maffei VJ, Zaneveld JR, Yurgel SN, Brown JR, Taylor CM, et al. PICRUSt2 for prediction of metagenome functions. Nat Biotechnol. 2020;38(6):685-8.

6. McMurdie PJ, Holmes S. phyloseq: an R package for reproducible interactive analysis and graphics of microbiome census data. PLoS One. 2013;8(4):e61217.

7. Dixon P. VEGAN, a package of R functions for community ecology. Journal of vegetation science. 2003;14(6):927-30.

8. Anderson MJ. Permutational multivariate analysis of variance (PERMANOVA). Wiley statsref: statistics reference online. 2014:1-15.

9. Mallick H, Rahnavard A, McIver LJ, Ma S, Zhang Y, Nguyen LH, et al. Multivariable association discovery in population-scale meta-omics studies. PLoS computational biology. 2021;17(11):e1009442.

10. Price MN, Dehal PS, Arkin AP. FastTree: computing large minimum evolution trees with profiles instead of a distance matrix. Mol Biol Evol. 2009;26(7):1641-50.

11. Katoh K, Rozewicki J, Yamada KD. MAFFT online service: multiple sequence alignment, interactive sequence choice and visualization. Brief Bioinform. 2019;20(4):1160-6.
